# Hydrogen Sensing Mechanism of WS_2_ Gas Sensors Analyzed with DFT and NAP-XPS

**DOI:** 10.3390/s23104623

**Published:** 2023-05-10

**Authors:** Tomoya Minezaki, Peter Krüger, Fatima Ezahra Annanouch, Juan Casanova-Cháfer, Aanchal Alagh, Ignacio J. Villar-Garcia, Virginia Pérez-Dieste, Eduard Llobet, Carla Bittencourt

**Affiliations:** 1 Department of Materials Science, Graduate School of Science and Engineering, Chiba University, 1-33 Yayoi-cho, Inage-ku, Chiba-shi 263-8522, Chiba, Japan; tomoya_2000@chiba-u.jp (T.M.); pkruger@chiba-u.jp (P.K.); 2 Departament d’Enginyeria Electronica, Universitat Rovira i Virgili, Països Catalans 26, 43007 Tarragona, Spaineduard.llobet@urv.cat (E.L.); 3 Chimie des Interactions Plasma Surface, CIRMAP, Université de Mons, Place du Parc 23, 7000 Mons, Belgium; juan.casanova@urv.cat; 4 ALBA Synchrotron Light Source, Carrer de la Llum 2-26, 08290 Cerdanyola del Vallès, Spain

**Keywords:** NAP-XPS, DFT, gas sensing, 2D materials, TMDCs

## Abstract

Nanostructured tungsten disulfide (WS_2_) is one of the most promising candidates for being used as active nanomaterial in chemiresistive gas sensors, as it responds to hydrogen gas at room temperature. This study analyzes the hydrogen sensing mechanism of a nanostructured WS_2_ layer using near-ambient-pressure X-ray photoelectron spectroscopy (NAP-XPS) and density functional theory (DFT). The W 4f and S 2p NAP-XPS spectra suggest that hydrogen makes physisorption on the WS_2_ active surface at room temperature and chemisorption on tungsten atoms at temperatures above 150 °C. DFT calculations show that a hydrogen molecule physically adsorbs on the defect-free WS_2_ monolayer, while it splits and makes chemical bonds with the nearest tungsten atoms on the sulfur point defect. The hydrogen adsorption on the sulfur defect causes a large charge transfer from the WS_2_ monolayer to the adsorbed hydrogen. In addition, it decreases the intensity of the in-gap state, which is generated by the sulfur point defect. Furthermore, the calculations explain the increase in the resistance of the gas sensor when hydrogen interacts with the WS_2_ active layer.

## 1. Introduction

Global warming is a challenge for humans. By the 4th of November 2016, 194 parties worldwide had signed a declaration of intent to substantially reduce global greenhouse gas emissions and limit the global temperature increase this century to 2 degrees Celsius with the Paris Agreement [1]. The European Union has set a rather aggressive goal of reducing greenhouse gases by 55% by 2030 [2]. Carbon dioxide, which accounts for approximately 80% of greenhouse gases [3,4,5], must be reduced to achieve those goals. In this context, hydrogen has been attracting attention as a clean energy source due to its advantage of non-toxic by-product generation when burned [6]. However, since hydrogen has low ignition energy and high flammability [7], requires storage under high pressure, and triggers metal embrittlement, the reliable and fast detection of hydrogen leaks is essential for its safe application.

Since the Paris Agreement, many countries have established policies and actions searching for alternative clean energy to limit the CO_2_ footprint and achieve net-zero emissions by 2050 [8,9]. However, reaching the complete decarbonization of the global economy will require diversifying renewable energy sources. Among other emerging technologies, sustainable production of green hydrogen in large quantities has the potential to serve as an energy carrier at the core of a carbon neutral system of energy production and use. Indeed, hydrogen is abundant, 100% sustainable, storable, and easy to transport.

The industry that uses hydrogen as a feedstock is well established. It had a total estimated value of USD 155 billion in 2022 and is expected to grow significantly in the coming years. The rising focus on cleaner energy and favorable government regulations are encouraging. Therefore, market growth is expected to be larger than the present-day 10% per year.

Successfully deploying green hydrogen technology requires using affordable yet reliable hydrogen sensors throughout the whole chain from generation, transport/distribution, storage, and consumption. On the one hand, hydrogen sensors are needed at generation to help monitor and optimize the efficiency of the hydrogen evolution reaction (HER). However, on the other hand, hydrogen sensors are also required to ensure the safe operation of the whole hydrogen chain. Indeed, H_2_ is a small molecule that easily diffuses through materials and may leak from containers, especially when kept at high pressure (e.g., 900 bar). In addition, hydrogen is odorless and collects under roofs and overhangs, where it can generate explosion hazards, as H_2_ is flammable in the air when its concentration lies in the 4% to 75% range.

Recent gas sensors have a variety of response mechanisms [10,11,12,13,14], and the chemiresistive gas sensor is one of the most widely used gas sensing devices due to its advantages, such as the ease of fabrication, simplicity of operation, low cost, and moderate power consumption [15,16]. Currently, commercially available hydrogen sensors consist of catalytic, electrochemical, and, to a lesser extent, chemiresistive metal oxide gas sensors. Catalytic sensors are operated at elevated temperatures (>600 °C) and suffer from a complete lack of selectivity, as any combustible gas generates a response. Electrochemical sensors use a liquid and are hard to miniaturize. Furthermore, hydrogen electrochemical sensors suffer from important cross-sensitivity to alcohols. Finally, commercially available metal oxide hydrogen sensors consist of a thick film of an n-type metal oxide (e.g., SnO_2_, In_2_O_3_) loaded with Pt or Pd nanoparticles. The main drawbacks of metal oxide sensors are their lack of selectivity, the relatively high operating temperatures (200 to 400 °C), and the long-term response drift. In addition, thick-film metal oxides require a significant amount of noble metal loading, which increases cost. Despite the pitfalls, chemiresistive gas sensors have an important share of the global sensor market (23.4% and USD 1.1 billion in 2022).

As gas-sensitive materials in chemiresistive gas sensors, transition metal dichalcogenides (TMDCs) are promising candidates. TMDCs are a group of materials with the formula MX_2_, where M is a transition metal (such as Ti, V, Mo, and W) and X is a chalcogen (S, Se, or Te) [17]. The transition metal M and the chalcogen X form a layered structure, and the layers are stuck by a weak van der Waals force [17]. This morphology brings a large surface-to-volume ratio [15,16,18], enhancing the active surface interaction with target gases. In addition, gas sensors using TMDC materials show good potential for low operating temperature gas-sensing applications. Even room temperature detection has been reported, which helps achieve ultra-low power gas sensing [15,16,18]. For these reasons, TMDCs have been investigated as active materials for several years [15], with MoS_2_ and WS_2_ as the two most studied TMDCs. While MoS_2_ has been reported mainly as useful for detecting NO_2_ and NH_3_ [19], WS_2_ has been found useful for detecting NH_3_ [20], NO_2_ [21,22,23], H_2_S [21,24,25], or H_2_ [26,27]. Therefore, WS_2_ is a good candidate material for detecting hydrogen at moderate operating temperatures or even at room temperature.

While the gas-sensing mechanism of metal oxides, by far the most used gas-sensitive material in chemiresistive gas sensors, is relatively well understood [18], the sensing mechanism in TMDCs has yet to be studied as deeply. Sensing mechanisms can involve many changes, such as charge transfer and doping, intercalation, and shifts in permittivity and lattice vibrations [17]. However, recent theoretical studies show that the gas sensing mechanisms of TMDCs and related layered materials have charge transfer processes as the main route for detecting gases [27,28,29,30], in which the sensing materials act as charge acceptors or donors [4,28,29,30]. For example, Changjie Zhou et al. reported that hydrogen adsorption brings a relatively small amount of charge transfer from the WS_2_ monolayer to the adsorbed hydrogen molecule [31]. However, such calculation results are more complex than the actual environment in which gas sensors operate. Recently, the development of near-ambient pressure X-ray photoelectron spectroscopy (NAP-XPS) has emerged as a new tool for gaining insight into the gas sensing mechanism of nanomaterials.

NAP-XPS has several advantages for analyzing the active surface of chemiresistive gas sensors. The first is that NAP-XPS can run measurements at elevated pressures, as the name suggests, thus enabling the dosage of gas mixtures while XPS is conducted. The second is that it enables measuring the in situ XPS spectra as the device operates, allowing an ‘operando’ study of gas sensing. Finally, although, like XPS, NAP-XPS cannot detect hydrogen directly, it can trace the changes in the spectra recorded on the sensor active layer caused by its exposure to hydrogen gas.

In this study, we analyze the hydrogen sensing mechanism of chemiresistive WS_2_ gas sensors using NAP-XPS measurements under ‘operando’ conditions and density-functional-theory (DFT)-based calculations to conduct a study from both experimental and theoretical aspects. NAP-XPS spectra of the WS_2_ active surface were measured as hydrogen was dosed in the analysis chamber, and the chemiresistive response was recorded. These studies were conducted for gas sensitive film operated at room and high temperatures.

## 2. Methods

### 2.1. Experimental Methods

#### 2.1.1. Material Synthesis

Tungsten disulfide films were directly grown onto silicon oxide substrates (SiO_2_) using a two-step deposition technique. In the first one, tungsten trioxide nanoneedles (WO_3_) were grown at 400 °C via aerosol-assisted chemical vapor deposition (AACVD) of tungsten hexacarbonyl (W(CO)_6_, 50 mg) dissolved in a mixture of 15 mL of acetone and 5 mL of methanol, as described earlier in [32,33]. After that, the obtained films were annealed at 500 °C for 2 h under synthetic air. In the next step, WO_3_ nanoneedles were subjected to a sulfurization process at 900 °C, using the atmospheric pressure CVD method (APCVD). Before the sulfurization process, the quartz tube was flushed with argon (Ar) to remove oxygen from the reactor. Next, 800 mg of sulfur was evenly distributed in two alumina boats and placed at different temperature zones (900 °C and 40 °C) to provide a sufficient sulfur environment by pushing the tube 5 cm toward high temperature after 30 min of deposition. It is worth noting that the substrate is kept at the 900 °C temperature zone. The room temperature was 22 °C.

#### 2.1.2. Sensor Connection

**Figure 1:**
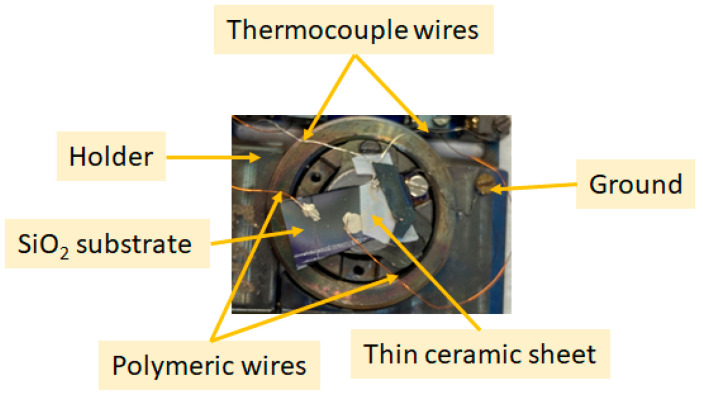
Photograph of the NAP-XPS sample holder.

A metallic holder (Figure 1) with five outputs was used to study the sensors’ active layer inside the NAP-XPS chamber. Two outputs were used to measure the film resistance changes in real time via the connection of two polymer-coated Pt wires between the holder and the SiO_2_ substrate. These connections were connected via vacuum sealed feedthroughs to the Agilent-34972A multimeter (Santa Clara, CA, USA). We have used a conductive silver paste to bond these polymer-coated Pt wires to the sensor material on the substrate. Moreover, two other outputs were employed to evaluate the temperature of the substrate by connecting thermocouple wires placed over the substrate (Figure 1). Finally, we used thin ceramic sheets to insulate the sample from the holder, as shown in Figure 1. So, the sample is grounded only through the Agilent-34972A multimeter outside the chamber, via the polymer-coated Pt wires.

#### 2.1.3. Gas Sensing Measurements

Gas sensing tests were performed using the gas delivery system. The target gas was hydrogen. The sample holder was introduced to the ultra-high vacuum NAP-XPS chamber and connected via vacuum-sealed feedthroughs to an Agilent-34972A multimeter to monitor real-time film resistance changes. Moreover, the heating was completed using a PID controlled infrared laser (λ = 808 nm) focused on a stainless-steel plate on top of which the sensor substrate with the sensor material was mounted. The temperature was monitored with a K-type thermocouple in direct contact with the substrate.

Sensing testing measurements were performed in our home laboratory before the beamtime at ALBA; the sensor was inserted in a Teflon chamber, which contains electrical contacts that allow real-time monitoring of the sensor resistance. The multimeter used for these measurements was identical to the one employed at ALBA. The sensor working temperature was obtained by applying a bias voltage to the sensor heating element via an external power supply. The gas from an H_2_ bottle (1000 ppm) was inserted in the testing Teflon chamber through mass-flow controllers. The purity of the hydrogen gas was 99.999%. Finally, the sensor response was calculated by using the following formula:

(1) $$R\left(\%\right)=\frac{Rgas-Rair}{Rair}*100$$

where *Rgas* is the value of the sensor resistance after being exposed for 10 min to a given gas/vapor diluted in air and *Rair* is the baseline resistance of the sensor exposed to dry air.

#### 2.1.4. Material Characterization

The morphology of the films was investigated using a field-emission scanning electron microscope Hitachi 2000 (Tokyo, Japan) and FEI Helios Nanolab 650 (Hillsboro, OR, USA), while their phase composition was analyzed via RAMAN spectroscopy, Renishaw in Via, laser 514 nm, ion argon-Novatech, 25 mW (Wotton-under-Edge, England, UK).

Near Ambient Pressure Photoelectron spectroscopy (NAP-XPS) was used to study the chemical response of the sensor active layer when exposed to hydrogen at room temperature and at 150 °C. The NAP-XPS endstation is installed at the NAPP branch of the CIRCE beamline in the ALBA synchrotron light source (Barcelona, Spain) [34]. This endstation consists of a load lock chamber, a preparation chamber, and an µ-metal shield analysis chamber for XPS measurements in the pressure range of UHV up to 20 mbar. A pressure difference of 10^9^ between the detector and sample is obtained using a PHOIBOS 150 NAP analyzer (SPECS Berlin/Germany). A differentially pumped photon beam entrance allows working at pressures in the millibar range without using a membrane for vacuum protection between the analysis chamber and the beamline.

An IR laser (with up to 150 W power) installed outside the analysis chamber mounted directly on a viewport with a protection cover allows heating the sample to temperatures above 1000 °C, in UHV and 750 °C at the millibar pressure range. The laser beam comprises a matrix of 19 individual beamlets in a hexagonal array allowing homogenous heating of the sample surface. At a 5 cm distance, the laser beam diameter at the sample position is 9 mm.

The photon excitation during the NAP-XPS analysis was 850 eV. The survey spectra were acquired with a step energy of 1 eV and pass energy of 20 eV, while the core level spectra had a step energy of 0.1 eV and pass energy of 10 eV. We analyzed the XPS measurements using Casa XPS (Devon, UK) [35] software to fit XPS spectra. Four regions were analyzed using NAP-XPS: W 4f, S 2p, O 1s, and C 1s regions. The background was removed using Shirley’s method. The C 1s peak position was used to calibrate the XPS spectra, and the C-C peak was set to 284.8 eV for the calibration. To fit the W 4f doublet peak, we imposed the following constraints: the intensity ratio between the 4f_5/2_ and 4f_7/2_ components was set to 0.75, the full width at half maximum (FWHM) of both components was the same, and the splitting energy due to spin–orbit correlation was fixed as 2.17 eV [36]. For the S 2p doublet fitting, the intensity ratio between the 2p_1/2_ and 2p_3/2_ components was set to 0.511, following the experimental literature [37,38,39]. The FWHM of both components was the same value, and the spin–orbit splitting energy was set to 1.16 eV. We restrained the FWHM of the peaks to be less than 2.5 eV. We calculated the relative concentration of each element in the WS_2_ film from the XPS survey spectra.

### 2.2. Theoretical Methods

To support the experimental results, the interaction of H_2_ with the WS_2_ surface was modeled using Density Functional Theory (DFT) with the Perdew–Burke–Ernzerhof (PBE) exchange-correlation functional including van der Waals corrections in the DFT-D3 scheme [40]. All calculations were conducted with the plane-wave projector augmented wave method VASP (Vienna Ab initio Simulation Package) [41]. The valence electrons for tungsten, sulfur, hydrogen, and oxygen were chosen as 5d^4^6s^2^, 3s^2^3p^4^, 1s, and 2s^2^2p^4^, respectively. The energy and force convergence criteria were set to 10^−5^ eV and 0.02 eVÅ^−1^), respectively. An energy cut-off of 500 eV and a Γ-centered 4 × 4 × 1 k-point mesh were used for the 3 × 3 surface cell of WS_2_.

Tungsten disulfide is a TMDC with a layered structure where the hexagonal WS_2_ monolayers are held together by weak van der Waals interaction. We consider the stable 2H-WS_2_ phase (space group P6_3_/mmc) with lattice constants a = 9.459 Å [42].

**Figure 2:**
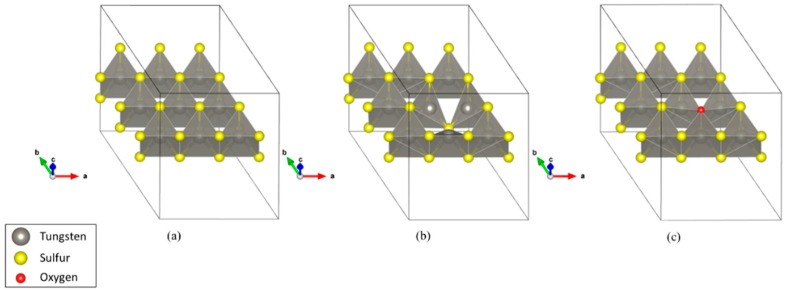
The three WS_2_ structures considered in the DFT calculation: (**a**) A defect-free WS_2_ monolayer, (**b**) a WS_2_ monolayer with one sulfur point defect, and (**c**) a WS_2_ monolayer in which one sulfur atom is replaced by one oxygen atom.

We considered three systems for hydrogen adsorption on the WS_2_ (0001) surface, namely a defect-free WS_2_ monolayer, a WS_2_ monolayer with one sulfur point defect, and a WS_2_ monolayer in which one sulfur atom is replaced with one oxygen atom as shown in Figure 2. A 3 × 3 supercell was used. To model the WS_2_ surface, we inserted 16 Å of vacuum between repeated slabs. Figure 2 shows the systems after structural optimization.

**Figure 3:**
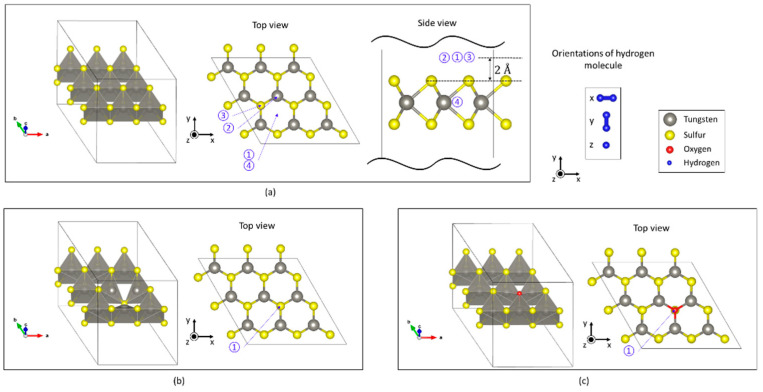
Adsorption geometries. For each adsorption site, three orientations of hydrogen, along the *x*-, *y*-, and *z*-axes, were simulated: (**a**) four adsorption sites (1–4) on the defect-free WS_2_ monolayer, (**b**) adsorption site above the sulfur point defect, and (**c**) adsorption site above WS_2_ with oxygen replacing one surface atom.

The change in the resistance of the WS_2_ gas sensor would be caused by physical or chemical adsorption of hydrogen on the WS_2_ monolayers, so we focus on the interaction between the optimized WS_2_ monolayers and a hydrogen molecule. The adsorption energy is defined as follows:

(2) $$E_{ad}=E_{adsorbedsystem}-\left(E_{monolayer}+E_{targetmolecule}\right)$$

where $E_{ad}$ is adsorption energy, $E_{adsorbedsystem}$ is the total energy of an adsorbed system, $E_{monolayer}$ is the total energy of the monolayer, and $E_{targetmolecule}$ is the total energy of a target molecule. Under this definition, the adsorbed system is stable if the adsorption energy is negative. When the adsorption energy is positive, the adsorbed system is unstable, and the adsorption of the target molecule is impossible (at the considered adsorption site). We simulated the adsorption of one hydrogen molecule at several sites in each of the three surface models, as shown in Figure 3. In the structural optimization, several initial configurations were chosen according to the adsorption sites and the orientation of a hydrogen molecule. For example, on the defect-free WS_2_ monolayer, we chose four different sites, as shown in Figure 3a. On the WS_2_ monolayer with one sulfur defect, we chose the site above the sulfur point defect (Figure 3b). For the adsorption site on the WS_2_ monolayer containing one oxygen, we chose the site above the oxygen atom (Figure 3c). In all cases, we simulated three hydrogen orientations along the *x*-, *y*-, and *z*-axes, as shown in Figure 3. After structural optimization of each atomic position, each adsorption energy was calculated using Equation (2).

## 3. Results

### 3.1. Experimental Results

#### 3.1.1. Characterization of the Materials

**Figure 4:**
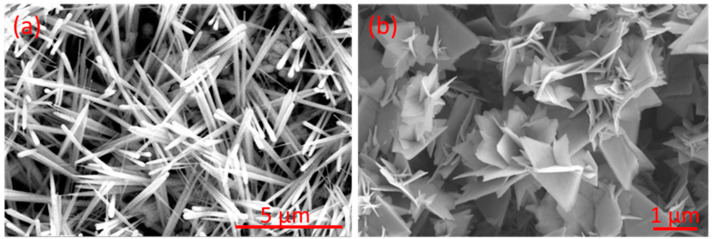
FESEM images of (**a**) WO_3_ nanoneedles and (**b**) WS_2_ nanotriangles.

Tungsten trioxide nanoneedle films were successfully grown via AACVD at 400 °C onto silicon oxide substrates. These films were characterized by homogenous coverage over the substrate, strong adhesion, and dark blue color, which changed to light green after the annealing. Figure 4a shows the morphology of the grown nanoneedle films. As we can see, nanoneedles are interconnected. Their diameter is less than 100 nm, and their length is about 7 μm [32,33]. These films were subject to a sulfurization process to obtain WS_2_. Figure 4b depicts the morphology of the obtained tungsten disulfide material (i.e., resulting from the sulfurization of WO_3_ nanoneedle films). It is composed of nanotriangles that were assembled in a three-dimensional orientation. The thickness of the WS_2_ films depends on the length of the WO_3_ nanoneedles grown by the AACVD process. Nanoneedle length is controlled by the temperature, flow rate, and quantity of precursor used. The thickness of the films reported here was 10 μm ± 1 μm. It is worth mentioning that the color of the films after sulfurization converts to black.

**Figure 5:**
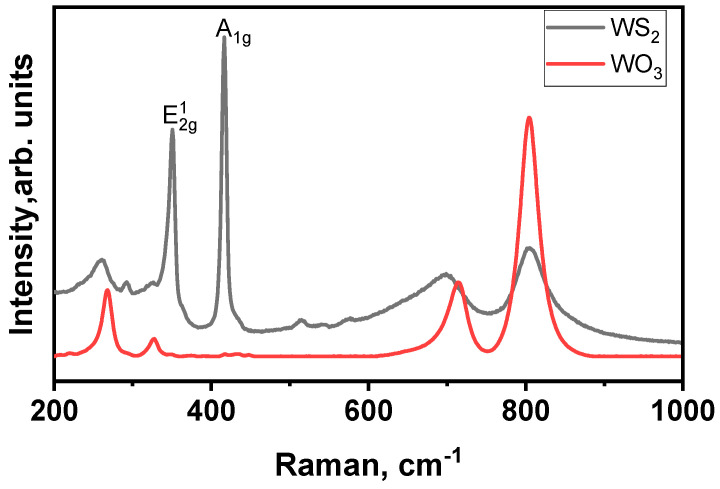
Raman spectra of WO_3_ and WS_2_ nanomaterials.

Figure 5 represents the Raman spectra obtained from WO_3_ and WS_2_ samples. This technique gives us insights regarding the phase composition of the films, their crystal quality, and the number of layers that compose our TMDs material. Figure 5 indicates the spectrum of WO_3_. It has four peaks—269 cm^−1^, 326 cm^−1^, 714 cm^−1^, and 804 cm^−1^—that demonstrate the presence of a monoclinic tungsten trioxide phase, in line with our previously reported works [32,43].

Regarding the WS_2_ spectrum (Figure 5), it is composed of two intense Raman peaks indicative of the formation of 2H-WS_2_. These peaks are located at 350 ($E_{2g}^{1}$) and 417 cm^−1^ ($A_{1g}$). The first mode corresponds to the in-plane vibration of W and S atoms, and the second one indicates the vibration of sulfides in the out-of-plane direction [21,44]. Therefore, the ratio of their relative intensity reveals the formation of multilayer WS_2_. Moreover, it has two peaks at 700 cm^−1^ and 805 cm^−1^, indicating the presence of WO_3_ impurities; their relative intensities are much lower than those in the WO_3_ spectrum.

#### 3.1.2. Characterization of the Sensors

**Figure 6:**
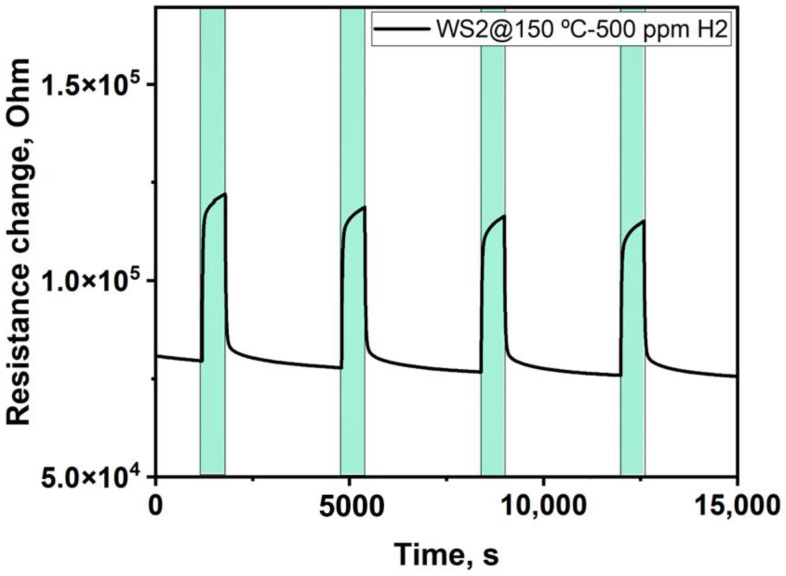
Sensor resistance changes to 500 ppm of H_2_ as a function of time.

The functionality of tungsten disulfide gas sensors towards the detection of hydrogen (H_2_) was assessed before measuring them at the Alba synchrotron. For this purpose, we exposed them to 500 ppm of H_2_ at their optimal working temperature of 150 °C. The exposure time was 10 min, and the recovery to the baseline resistance was 1 h. Figure 6 represents typical sensor resistance changes towards H_2_ as a function of time. The WS_2_ sensor behaves as a p-type semiconductor, increasing the resistance while exposed to a reducing gas and decreasing it while cleaning with dry air. The response drops by about 15% when the humidity is raised from 3% to 50%, as reported before [21,24]. Moreover, the sensor showed good sensitivity to H_2_; the response to 500 ppm of H_2_ was 34%. Additionally, the responses were fast, stable, and reproducible; after each H_2_ exposure, the sensor quickly returned to its initial baseline resistance.

#### 3.1.3. NAP-XPS

**Figure 7:**
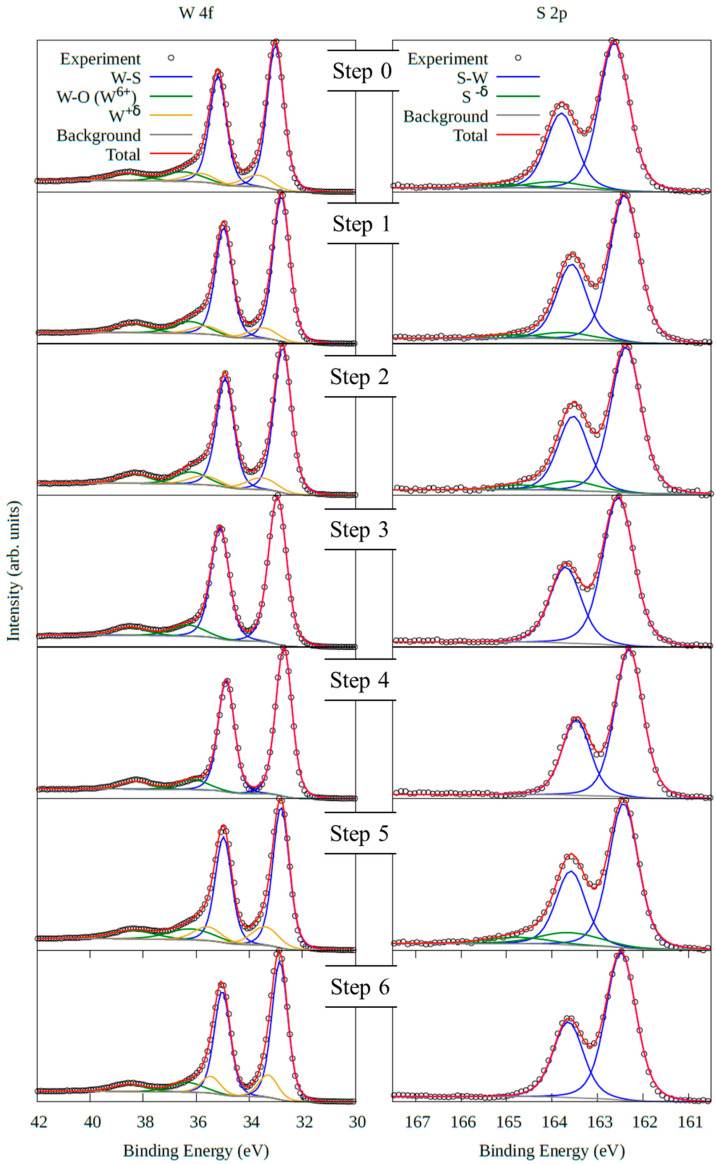
NAP-XPS spectra of W 4f and S 2p for the WS_2_ active surface gas sensor. The measurements were performed under continuous changes in the environmental condition of the chamber through steps 0 to 3 and 4 to 6.

NAP-XPS was used to analyze the interaction between hydrogen gas and the WS_2_ active surface gas sensor. The WS_2_ active surface was placed at the focal position of the NAP-XPS analyzer, and the XPS spectra were recorded continuously as the concentration of hydrogen gas changed. The resistance of the active layer was measured simultaneously with the XPS spectra. We focus on the difference in the sensing response at room temperature and 150 °C. The measurement sequence is divided into two series as shown in Table 1: the first series, the sensor operates at room temperature (at step 0, the WS_2_ gas sensor was inserted in UHV; step 1, in 1000 ppm H_2_ gas; step 2, in 5000 ppm H_2_ gas; step 3, in UHV), and the second series at higher temperatures with the sensor device heated at 150 °C (step 4, the WS_2_ gas sensor was in UHV; step 5, in 1000 ppm H_2_ gas; step 6, in UHV). Figure 7 shows the NAP-XPS spectra analysis of the W 4f, S 2p peaks recorded in each step. In step 0, the fitting analysis of the W 4f spectrum shows two intense components at 33.0 eV and 35.2 eV assigned to the W 4f doublet in WS_2_ [21,45] and components at 36.5 eV and 38.7 eV, to W 4f in WO_3_ [21,45]. The components at 33.7 eV and 35.9 eV would be assigned to the W 4f doublet in a mixed oxidation state of tungsten (denoted W^+δ^ in the following). The W 4f spectra in steps 1 to 3 and 8 to 10 are assigned similarly. Furthermore, the spectrum recorded in the S 2p region shows an intense doublet with components centered at 162.6 eV and 163.8 eV which can be assigned to S 2p in WS_2_ [21,45]. The other doublet at 163.9 eV and 165.1 eV may be assigned to S 2p in mixed oxidation states of sulfur (denoted S^−δ^). The S 2p NAP-XPS spectra through steps 1 to 6 are similarly fitted.

First, we focus on the change in the spectra through steps 0 to 3, i.e., the sensor was exposed to H_2_ at room temperature. Adding H_2_ gas in steps 1 and 2 did not change the components’ ratios and shapes. However, the resistance of the WS_2_ gas sensor changed through steps 0 to 2, from 110 kΩ to 145 kΩ (Table 1). This suggests that the change in resistance is caused not by chemical adsorption but by the physical adsorption of hydrogen on the WS_2_ monolayers. Moreover, after the interaction with H_2_ and returning to UHV, the intensity of the fitting components associated with W^+δ^ and S^−δ^, which are the yellow line W 4f and the green line in S 2p, decreases, suggesting that the exposure of hydrogen leads to the reduction of the WS_2_ surface.

Next, we will turn to the analysis of the XPS spectra recorded through steps 4 to 6. The components associated with mixed oxides in W 4f (yellow line) and in S 2p spectra (green line) disappeared at the end of step 3 and reappeared after the introduction of H_2_ gas (from steps 4 to 5). These results show that hydrogen interacts with both tungsten and sulfur atoms in WS_2_ active surface. However, the yellow components (W4f) remained, while the green components (S 2p) disappeared after the H_2_ was evacuated from the NAP-XPS chamber and it returned to UHV. The variation in the intensity of the green and yellow components suggests that hydrogen interacts strongly with tungsten atoms making chemical bonds. In contrast, it makes weak bonds with sulfur atoms when interacting with the sensor heated at 150 °C.

The relative concentration of each element in WS_2_ film was evaluated from the NAP-XPS survey spectra: tungsten, sulfur, and oxygen. Table 1 shows the concentration of those elements in the WS_2_ sensor active surface, and the column of S/W shows the ratio of the sulfur to tungsten concentrations. It is seen that the relative concentrations of W and S hardly vary throughout the steps. However, the oxygen concentration changed. Thus, the active surface of WS_2_ is stable under hydrogen exposure and heating.

Some conclusions about the atomistic mechanism during hydrogen exposure can be drawn from the preceding analysis. First, hydrogen interacts with the WS_2_ surface; the W 4f yellow component and S 2p green component appeared in step 5 when WS_2_ was exposed to hydrogen gas, which shows that these components are directly related to hydrogen interaction with the WS_2_ surface. Since new components are needed for best fitting the W 4f and S 2p peaks, the introduced hydrogen interacts with tungsten and sulfur. However, the W4f yellow component remained after returning to the UHV environment, while the S 2p green component disappeared, as shown in step 6 (Figure 7). This suggests that hydrogen makes chemical bonds with tungsten atoms, but it does not make chemical bonds with sulfur atoms. To support these conclusions, we performed theoretical calculations as described in the next section.

### 3.2. Theoretical Results

#### 3.2.1. Stable Adsorption Sites on Each Monolayer

**Figure 8:**
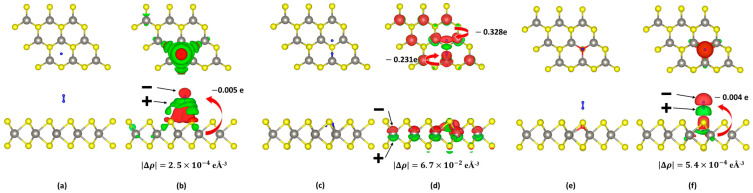
Most stable adsorbed systems for each monolayer. Top and side views of hydrogen adsorbed (**a**) on the defect-free WS_2_ monolayer (Figure 2a), (**c**) on the defect-containing WS_2_ monolayer (Figure 2b), and (**e**) on the oxygen-containing WS_2_ monolayer (Figure 2c). Panels (**b**,**d**,**f**) show those charge density differences after adsorption as isodensity surfaces. Red indicates a negative charge difference (i.e., an increase in electron density) and green indicates a positive value with each of the absolute isosurface values of (**b**) 2.5 × 10^−4^ eÅ^−3^, (**d**) 6.7 × 10^−2^ eÅ^−3^, and (**f**) 5.4 × 10^−4^ eÅ^−3^. The values of the charge transfer are indicated by the red arrows, respectively.

We simulated the adsorption of one hydrogen molecule on the different sites of the three WS_2_ surface structures shown in Figure 3. We found several stable adsorption configurations in all three cases. First, we focus on the adsorption of one hydrogen molecule on the defect-free WS_2_ monolayer. Then, we considered four different adsorption sites, namely above a W_3_S_6_ hexagon, on a sulfur atom and on a tungsten atom, as well as H_2_ intercalation, see the sites 1–4 in Figure 3. We found that the adsorption energies of three sites on the surface are negative, so adsorption is possible. In contrast, intercalation at site 4 is impossible because it has a very large positive adsorption energy of ~5 eV. The most stable adsorption configuration is the hydrogen molecule oriented along the *z*-axis and adsorbed at the hexagonal site, see Figure 8a. The adsorption energy is small (see Table 2), indicating physisorption. The height difference between the lower hydrogen atom and the upper sulfur layer (averaged over the three sulfur atoms) is 2.576 Å, and the corresponding average atomic distance is 3.155 Å. Furthermore, for the other two adsorption sites on the defect-free WS_2_ monolayer, the adsorbed hydrogen is most stable when the molecule is parallel to the *z*-axis. It makes physical sense that the hexagonal site is most stable because the number of nearest neighbor sulfur atoms is largest at this site and, thus, the H-S van der Waals interaction is maximal. Figure 8b shows the charge density difference and the charge transfer of the most stable system, i.e., the adsorption on the hexagonal site, as shown in Figure 8a. The charge density difference Δρ is defined as follows:

(3) $$\Delta \rho =\rho_{adsorbedsystem}-\left(\rho_{monolayer}+\rho_{targetmolecule}\right)$$

where $\rho_{adsorbedsystem}$, $\rho_{monolayer}$, and $\rho_{targetmolecule}$ are the charge density of the adsorbed system, the monolayer, and the target molecule, respectively. The distribution in Figure 8b shows that the hydrogen molecule is polarized by the interaction with the surface. We also calculated the charge transfer using Bader charge analysis [46]. The value of the charge transfer is −0.005 e from the monolayer to the hydrogen molecule, which is comparable to a previous calculation [31]. The value of the charge transfer is very small, but the result shows that hydrogen acts as an acceptor to the defect-free WS_2_ monolayer.

Second, we turn to the adsorption of one hydrogen molecule on the WS_2_ monolayer containing one sulfur point defect. We simulated three geometries of one hydrogen adsorption on the sulfur defect in the WS_2_ monolayer (see Figure 3b). Figure 8c shows the most stable adsorbed system whose adsorption energy is −299 meV. The adsorbed hydrogen molecule dissociates, and one hydrogen atom bonds with the nearest tungsten atom with a bond length of 1.698 Å, while the other hydrogen atom is attracted by the neighboring two tungsten atoms with an average distance of 1.858 Å. The hydrogen adsorption process is a dissociative chemisorption characterized by a sizeable adsorption energy, the splitting of the hydrogen molecule, and short H-W bond lengths. Figure 8d shows the charge density difference upon adsorption, calculated with Equation (2). The result in Figure 8d shows that the hydrogen adsorption on the sulfur point defect leads to a charge bias of the tungsten layer and an increase of charge around the hydrogen atoms. There is a charge transfer of −0.23 e and −0.33 e from the WS_2_ monolayer to the two hydrogen atoms, i.e., a total charge transfer of −0.56 e, see Figure 8d and Table 2. This value is quite large, indicating that hydrogen molecules can act as strong acceptors at the WS_2_ monolayer if they adsorb on the sulfur point defect. We note that for TMDC-metal interfaces, substantial charge redistribution has been reported both experimentally [47,48] and theoretically [49]. The actual charge transfer depends on the degree of oxidation and defect concentration, as we have found here in the case of hydrogen adsorption.

Third, we focus on hydrogen adsorption on the oxygen atom that has substituted a sulfur atom in the WS_2_ monolayer (see Figure 2c). We have considered three ways that one hydrogen adsorption could occur on the oxygen in the WS_2_ monolayer (see Figure 3c). All of them have negative adsorption energy. In the most stable configuration, the hydrogen molecule is parallel to the *z*-axis, as shown in Figure 8e, and the adsorption energy is −116 meV. The distance between the lower hydrogen atom and the oxygen is 2.367 Å. Figure 8f shows the charge density difference of the adsorbed system. The red (negative) and green (positive) isosurfaces illustrate that the adsorbed hydrogen is polarized by the oxygen atom, which gets a slightly negative bias. As a result, the charge transfer is negligible. Therefore, the adsorption process can be considered physisorption.

#### 3.2.2. The Density of States

**Figure 9:**
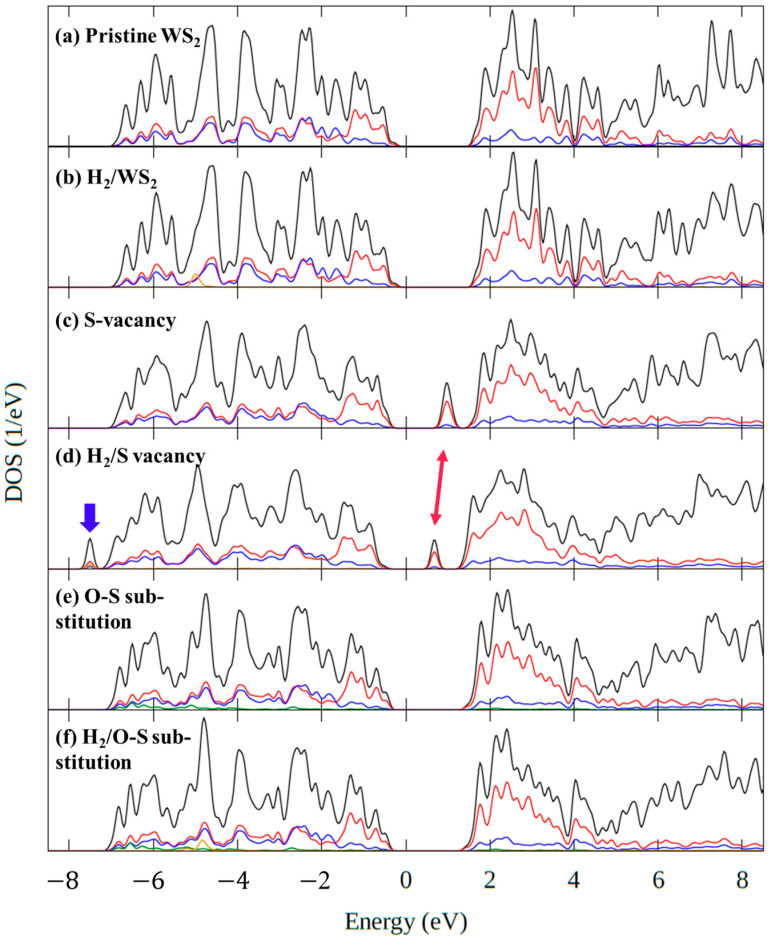
The density of states (DOS) of each system. The Fermi energy is at 0 eV. The black line is total DOS, and the other colored lines are partial DOS of each element. The DOS of (**a**) Pristine WS_2_ (Figure 3a), (**b**) H_2_/WS_2_ (Figure 8a), (**c**) S-vacancy (Figure 3b), (**d**) H_2_/S-vacancy (Figure 8c), (**e**) O-S substitution (Figure 3c), and (**f**) H_2_/O-S substitution (Figure 8e). The red arrow indicates the in-gap state and the blue arrow the new filled state.

We found several adsorption sites and stable systems. To analyze the electronic structure, we also calculated the DOS for these systems. First, we focus on how the electronic state changes when hydrogen adsorbs on the defect-free WS_2_ monolayer. Figure 9 shows the density of states (DOS) for different systems. The Fermi energy is taken as zero. Figure 9a shows the DOS of the defect-free WS_2_ monolayer, labeled as Pristine WS_2_. Figure 9b shows the DOS of the WS_2_ monolayer with a hydrogen molecule adsorbed on the center of a hexagonal site at the surface (labeled as H_2_/WS_2_), which is the most stable adsorbed system from the defect-free WS_2_ monolayer (see Figure 8a). The DOS of H_2_/WS_2_ is very similar to that of pristine WS_2_. Upon hydrogen adsorption, a shoulder appears at −4 eV, corresponding to H partial DOS (yellow line in Figure 9b), and the DOS shape changes slightly at 6 eV. However, the electronic states near the Fermi level remain unchanged. Therefore, hydrogen adsorption on a defect-free WS_2_ surface should not affect the electronic conduction of a defect-free WS_2_ monolayer.

Second, we examine how the electronic state changes when hydrogen adsorbs on a sulfur defect. Figure 9c shows the DOS of the WS_2_ monolayer with one sulfur defect (without hydrogen, Figure 2b) denoted as S-vacancy. Comparing the DOS of S-vacancy and pristine WS_2_, the main difference is that the sulfur defect gives rise to a new DOS peak in the band gap, about 1 eV above the Fermi level. This peak corresponds to two degenerate states per unit cell (i.e., per S vacancy) with a small band dispersion (about 0.2 eV). They are empty (acceptor) states, mainly containing 5d-orbitals of the W atoms nearest to the S-vacancy. So, in a chemical language, they are made by the dangling bonds of W. Figure 9d shows the DOS of WS_2_ with one hydrogen molecule adsorbed on the sulfur defect which we denote as H_2_/S-vacancy. Comparing H_2_/S-vacancy DOS and S-vacancy DOS, it is seen that intensity of the in-gap peak (indicated by the red arrow) is decreased by one half. Indeed, this peak has only one state per cell. The missing intensity is found in the new, filled state at −8.5 eV, below the valence band. This state is formed mostly by the 1s-orbital of the H atom which is symmetrically bonded to two W atoms (upper H atom in Figure 8d) as well as 5d orbitals of these two W atoms. The two electrons donated by the H_2_ molecule occupy this bonding W-H orbital. The second H-atom (lower in Figure 8d) forms a bond with only one W atom. This bonding is weaker, and the smaller charge transfer from W to H is minor (see Figure 8d). It does not give rise to a new peak in the DOS. Orbital analysis shows that the H-orbital is dispersed over to many states in the valence band. So, when H_2_ molecules are adsorbed on the S vacancies, half of the in-gap states are removed, which should have a substantial effect on the electron conduction of the WS_2_ monolayer. Indeed, when the number of in-gap acceptor states is reduced, fewer electrons can be thermally excited from the valence band, which leads to fewer charge carriers. Thus, H_2_ adsorption should cause an increase in the resistance of the WS_2_ monolayer in the WS_2_ gas sensor.

Third, we look at how the electronic states change when hydrogen adsorbs on an oxygen impurity atom replacing one sulfur atom in the WS_2_ monolayer (see Figure 2c). Figure 9e shows the DOS of the WS_2_ monolayer in which one sulfur atom is replaced with one oxygen atom labeled as O-S substitution DOS, and Figure 9f shows the DOS of the WS_2_ with one hydrogen molecule adsorbed on the oxygen denoted as H_2_/O-S substitution DOS. Again, there are no apparent changes around the Fermi level. However, the hydrogen adsorption on the oxygen impurity changes the shape of the DOS around 6 eV, and there is the addition of the partial DOS of the H_2_ around −5 eV. These minor changes are similar to the ones of adsorption on the defect-free WS_2_. Thus, hydrogen adsorption on a substitutional oxygen defect, should not affect the electronic conduction of the WS_2_ monolayer.

## 4. Conclusions

In summary, the hydrogen-sensing mechanism of a chemiresistive WS_2_ gas sensor has been studied using NAP-XPS and DFT calculation. Experiments were performed for different hydrogen concentrations at room temperature and 150 °C. Upon hydrogen adsorption, the resistance of the sensor increases in all cases. For the same H concentration, the relative increase is considerably larger at higher temperature than at room temperature, indicating stronger H-surface interaction. NAP-XPS spectra have been recorded during gas sensor operation, and we have traced the changes in the W4f and S2p spectra on the sensor’s active surface. The XPS spectral changes suggest that hydrogen is physisorbed on WS_2_ at room temperature, whereas at higher temperature hydrogen forms chemical bonds with tungsten (and not with sulfur). The much stronger H-surface interaction at higher temperature is in line with the larger change in resistance. We performed DFT calculations for H_2_ adsorption on WS_2_, considering three surface models, namely a pristine WS_2_ monolayer, a WS_2_ monolayer with a sulfur point defect, and one where a sulfur atom is replaced by oxygen. The hydrogen molecule interacts with the pristine WS_2_ surface through physisorption, which also occurs in the system where one sulfur atom is replaced with oxygen. In contrast, the hydrogen molecule dissociates on the sulfur vacancy, and the H atoms make chemical bonds with tungsten. In this case, the adsorption energy is the largest (in absolute value), and there is a sizeable charge transfer from the surface to the dissociated H atoms. The S vacancy gives rise to a narrow band of empty in-gap states, about 0.5 below the conduction band, with two degenerate states per vacancy. When H adsorbs on the vacancy, one of the two acceptor states becomes a W-H bonding state, which is filled by the electrons donated from H_2_. Consequently, the number of in-gap states is reduced by half, which should lead to an increase in resistivity. Since the S defect concentration is expected to increase with temperature, the theoretical results can explain the experimental findings. At high temperature, there are many S defects where hydrogen chemisorbs. This reduces the number of gap-states, leading to larger resistance. At room temperature, however, S vacancies are rare, and hydrogen is mostly physisorbed on the pristine WS_2_ surface which hardly changes the electronic structure near the Fermi level, and so the resistance change is much weaker.

## Figures and Tables

**Table 1:** WS_2_ gas sensor characteristics (resistance and chemical composition) for different ambient exposure and the concentrations of each element in the WS_2_ active surface, which are calculated from the NAP-XPS survey spectra in each step.

Steps	NAP-XPS Chamber Environment	R (kΩ)	W (%)	S (%)	O (%)	S/W
0	UHV at RT	110	34.8	52.1	13.1	1.5
1	1000 ppm H_2_ at RT	120	33.6	48.8	17.6	1.5
2	5000 ppm H_2_ at RT	145	32.6	49.6	17.8	1.5
3	UHV at RT	135	32.0	50.0	18.0	1.6
4	UHV with the device heating at 150 °C	80	33.5	48.8	17.7	1.5
5	1000 ppm H_2_ with the device heating at 150 °C	110	31.3	49.8	18.9	1.6
6	UHV with the device heating at 150 °C	75	27.1	42.7	30.2	1.6

**Table 2:** Adsorption energy and charge transfer (from WS_2_ to hydrogen) for each adsorption site.

Adsorption Site	Adsorption Energy (meV)	Charge Transfer (e)
Above a hexagon in defect-free WS_2_	−70	−0.005
On a sulfur defect	−299	−0.56
On an oxygen impurity	−116	−0.004

## Data Availability

Raw data are available from authors upon request.
